# Supplementary material for: Identification of photoperiod-induced specific miRNAs in the adrenal glands of Sunite sheep (Ovis aries)
Source: Front Vet Sci. 2022 Jul 22;9:888207. doi: 10.3389/fvets.2022.888207 (PMC9354845; doi:10.3389/fvets.2022.888207)
Supplement: Supplementary file 1 [file Data_Sheet_1.ZIP › Supplementary file/miRDeep2 file.zip!/miRDeep2 file/Know_Structure/SPLP42A2/oar-mir-380.pdf]

miRBase precursor : oar-mir-380
Total read count : 2920
oar-miR-380-5p read count : 40
oar-miR-380-3p read count : 2878
remaining reads : 2

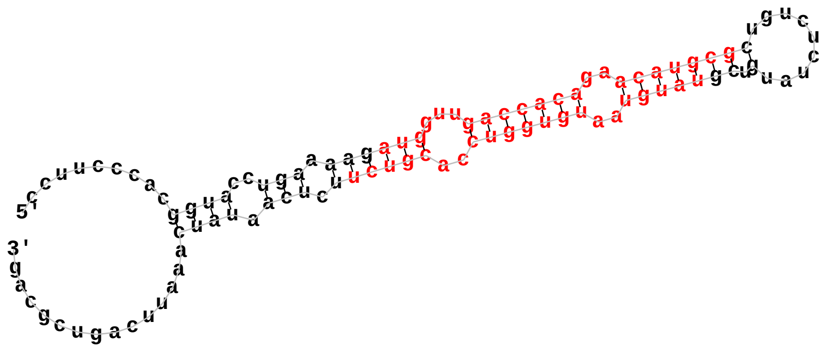

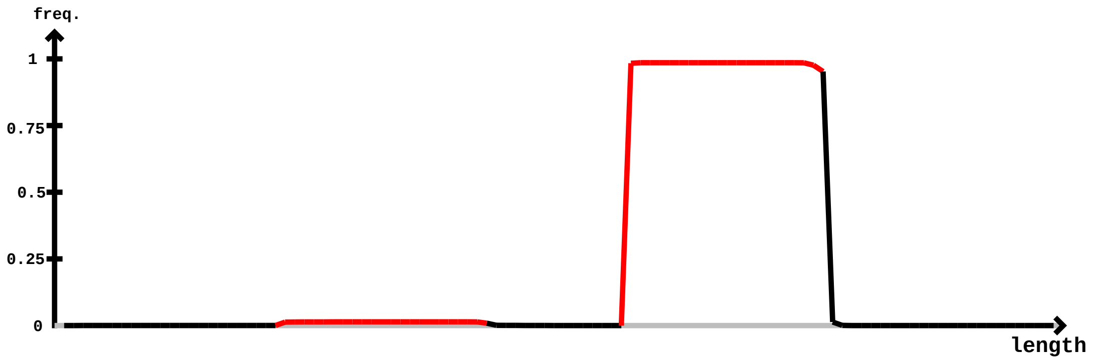

oar-miR-380-5p

oar-miR-380-3p

```
5'- ccuucccacgguaccugaaaagaugguugaccacagaacaugcgcugucucuaugucguauguaaugugguccacgucuucucaauaucaaauucagucgcag  -3'    exp
    .........((((..(((.((((((...(((((((..((((((((.........).)))))))..)))))))..)))))).))).)))).............   reads     mm        sample
    .cuucccacgguaccugaaaag.................................................................................   1         0         seq
    ......................augguugaccacagaacaug.............................................................   1         0         seq
    ......................augguugaccacagaacaugc............................................................   13        0         seq
    ......................augguugaccacagaacaugcg...........................................................   23        0         seq
    ......................augguugaccacagaacaugcgc..........................................................   1         0         seq
    .......................ugguugaccacagaacaugc............................................................   1         0         seq
    ........................gguugaccacagaacaugcgcug........................................................   1         0         seq
    ...........................ugaccacagaacaugcgcugucu.....................................................   1         0         seq
    ..........................................................uauguaaugugguccacg...........................   1         0         seq
    ..........................................................uauguaaugugguccacgu..........................   26        0         seq
    ..........................................................uauguaaugugguccacguc.........................   68        0         seq
    ..........................................................uauguaaugugguccacgucu........................   2738      0         seq
    ..........................................................uauguaaugugguccacgucuu.......................   37        0         seq
    ..........................................................uauguaaugugguccacgucuuc......................   2         0         seq
    ...........................................................auguaaugugguccacguc.........................   1         0         seq
    ...........................................................auguaaugugguccacgucu........................   5         0         seq
```
